# Network-pharmacology-based validation of TAMS/CXCL-1 as key mediator of XIAOPI formula preventing breast cancer development and metastasis

**DOI:** 10.1038/s41598-017-15030-3

**Published:** 2017-11-06

**Authors:** Neng Wang, Yifeng Zheng, Jiangyong Gu, Youli Cai, Shengqi Wang, Fengxue Zhang, Jianping Chen, Honglin Situ, Yi Lin, Zhiyu Wang

**Affiliations:** 1 The Research Centre for Integrative Medicine, Guangdong Provincial Academy of Chinese Medical Sciences, Guangzhou University of Chinese Medicine, Guangzhou, Guangdong, China; 2 Integrative Research Labrotary of Breast Cancer, Discipline of Integrated Chinese and Western Medicine, The second affiliated hospital of Guangzhou University of Chinese Medicine, Guangzhou, Guangdong, China; 3 Post-Doctoral Research Center, Guangzhou University of Chinese Medicine, Guangzhou, Guangdong, China; 4 School of Chinese Medicine, the University of Hong Kong, Pok Fu Lam, SAR Hong Kong

## Abstract

Network pharmacology has become a powerful means of understanding the mechanisms underlying the action of Chinese herbs in cancer treatment. This study aims to validate the preventive effects and molecular mechanisms of a clinical prescription XIAOPI formula against breast cancer. *In vivo* breast cancer xenograft data showed that XIAOPI delayed breast cancer development and efficiently inhibited lung metastasis, accompanied by prolonged survival benefits and decreased cancer stem cell subpopulations. However, similar phenomenon were not observed in a cell model. The herb-ingredient-target network analysis further identified a total of 81 genes closely correlated with the breast cancer chemoprevention effects of XIAOPI. Cytokine array analysis further validated CXCL-1 as the key target of XIAOPI both *in vitro* and *in vivo*. Evaluation of the mechanism demonstrated that CXCL-1 administration significantly abrogated the metastatic inhibition effects of XIAOPI on breast cancer migration, invasion, stem cells subpopulations, epithelial-mesenchymal transition(EMT), or mammosphere formation abilities. Overall, our study provides experimental evidence and molecular mechanisms that may facilitate the safe and effective use of herbal medicine for the prevention of breast cancer growth or metastasis, and may lead to CXCL-1-based therapeutic strategies for mammary malignancies.

## Introduction

Breast cancer is the most common malignancy in U.S. women, accounting for nearly one in three cancers. In 2017, it was estimated that there would be more than 2.8 million women with a history of breast cancer in the U.S.^1^. New cases of breast cancer reached 255,180 and mortality as high as 41,070^2^. Breast cancer is also one of the most common diagnosed cancers among women in 140 of 184 countries worldwide^3^. For example, breast cancer alone is expected to account for 15% of all new cancers in women in China. It was estimated that China had 272,400 new cases and 70,700 deaths of breast cancer in 2015^1^. From 2000 to 2015, the incidence of breast cancer in China has increased steadily^4^. Finding a way to prevent breast cancer and reduce its mortality has become an urgent issue worldwide.

Currently breast cancer prevention mainly includes three strategies: lifestyle modification, chemoprevention, and prophylactic surgery. Although alcohol consumption, obesity, and hormone replacement therapy have been evaluated as independent risk factors for breast cancer, there is no specific guideline for dietary or physical activity intervention specifically to prevent breast cancer^5^. Because of the lack of strong evidence and high-quality clinical trials, lifestyle adjustments are only recommended to patients as a supplemental measure in clinical settings. It is reasonable to encourage women to maintain an ideal body weight by restricting fat intake and undertaking 30–45 minutes of vigorous physical exercise three to five times per week^6^. Chemoprevention describes pharmacological intervention to reverse, suppress, or inhibit carcinogenic transformation. Selective ER modifiers (SERMs) and aromatase inhibitors (AIs) have been approved for clinical prevention^7^. Tamoxifen reduces ER-positive breast cancer risk by 62% but does not affect ER-negative breast cancer^8^. Raloxifene, a second-generation SERM, was found to reduce the risk of breast cancer by 44–76% in postmenopausal women^9^. Multiple clinical trials have demonstrated that AIs were capable to reduce breast cancer risk in postmenopausal women. For example, exemestane was confirmed to reduce the risk of breast cancer by 65%, but these protective effects were only observed in ER-positive cases^10^. Although SERMS and AIs are recommended as chemoprevention agents in clinical settings, their adverse events render their clinical use problematic. Patients suffer incidence risks of endometrial cancer, stroke, venous thromboembolism, musculoskeletal pain, osteoporosis, and even bone fracture^11^. With regard to prophylactic surgery, bilateral salpingoophorectomy and mastectomy are recommended to high-risk patients, especially those who carry the BRCA1/2 mutation^12^. Numerous retrospective and cohort studies have suggested that prophylactic mastectomy could reduce the risk of breast cancer by more than 90%^5^. However, mastectomy does not completely eliminate the risk of breast cancer because it is impossible to eradicate all terminal duct-lobular units in many women. In addition, 8–64% of post-surgical women experience one or more complications such as bleeding, infection, and skin flap necrosis^13^. Over 30% of patients report feeling embarrassed about the appearance of their breasts even after mammary reconstruction and difficulties in sexual arousal^14^. It is still necessary to find novel and safe prevention strategies.

Traditional Chinese Medicine (TCM) has a unique role in cancer prevention. In TCM philosophy, cancer is caused by the disturbances in endogenous physical condition and exogenous pathogenic factors. The disharmony of the body-mind communication network may also trigger cancer development^15^. For this reason, TCM doctors view cancer as a systemic disease and focus on holistic enhancement of inner defenses and restoration of normal balance for cancer therapy, which differs from Western medicine, which focuses only on killing cancer cells. TCM is appreciated in China’s rural areas and well-developed cities because of its 5000-year-old history and well-established theoretical approach. In a cross-sectional study conducted by the government of Taiwan, among 70,012 female breast cancer patients, 35.6% used TCM^16^. Several formulas have been developed into commercial products and applied in the tumor remission or stabilization stage. Examples of such treatments include Kanglaite injection, compound Kushen injection, Elemene emulsion injection, and Jinlong capsules, and they have shown sound clinical efficacy in improving overall survival, reducing toxicity, and preventing disease progression^15,17^. A great deal of effort has gone into understanding the therapeutic principles and molecular targets of herbal medicine in cancer prevention and treatment. Many TCM formulas or single active components have been reported to inhibit a variety of processes in cancer cell growth, invasion, and metastasis by modulating a wide range of molecular targets, including cyclooxygenase-2 (COX-2), nuclear factor-κB (NF-κB), and nuclear factor erythroid 2 -related factor 2 (Nrf2)-mediated antioxidant signaling pathways^18^. However, the mechanisms underlying most formulas remain unclear due to the complex composition of these medicines, which means several targets may be involved in its action.

In order to determine the holistic way of TCM from a molecular to system level, a variety of constructive technologies have been developed over the past few decades. Representative technologies include metabolomics, serum pharmacokinetics, proteomics, and genomic arrays^19^. These methodologies tend to focus on component identification, drug metabolism, and target screening. A more holistic method is needed to analyze the correlations associated with the herb-compound-target network. Recently, system biology has emerged as a novel tool that can be integrated with pharmacology. System pharmacology has made a significant contribution to investigation of the holistic overview of TCM by pharmacokinetic evaluation (absorption, distribution, and metabolism), target prediction, and network analysis^20^. By using the method, it may be possible to shift the single “one drug, one target” model to a more complex “drug-target network interaction” strategy^21^. Meanwhile, system pharmacology has greatly accelerated the drug target screening process. By combing disease target database and molecular validation, it is becoming much easier to explore the precise molecular target and mechanisms of TCM formulas.

**Figure 1:**
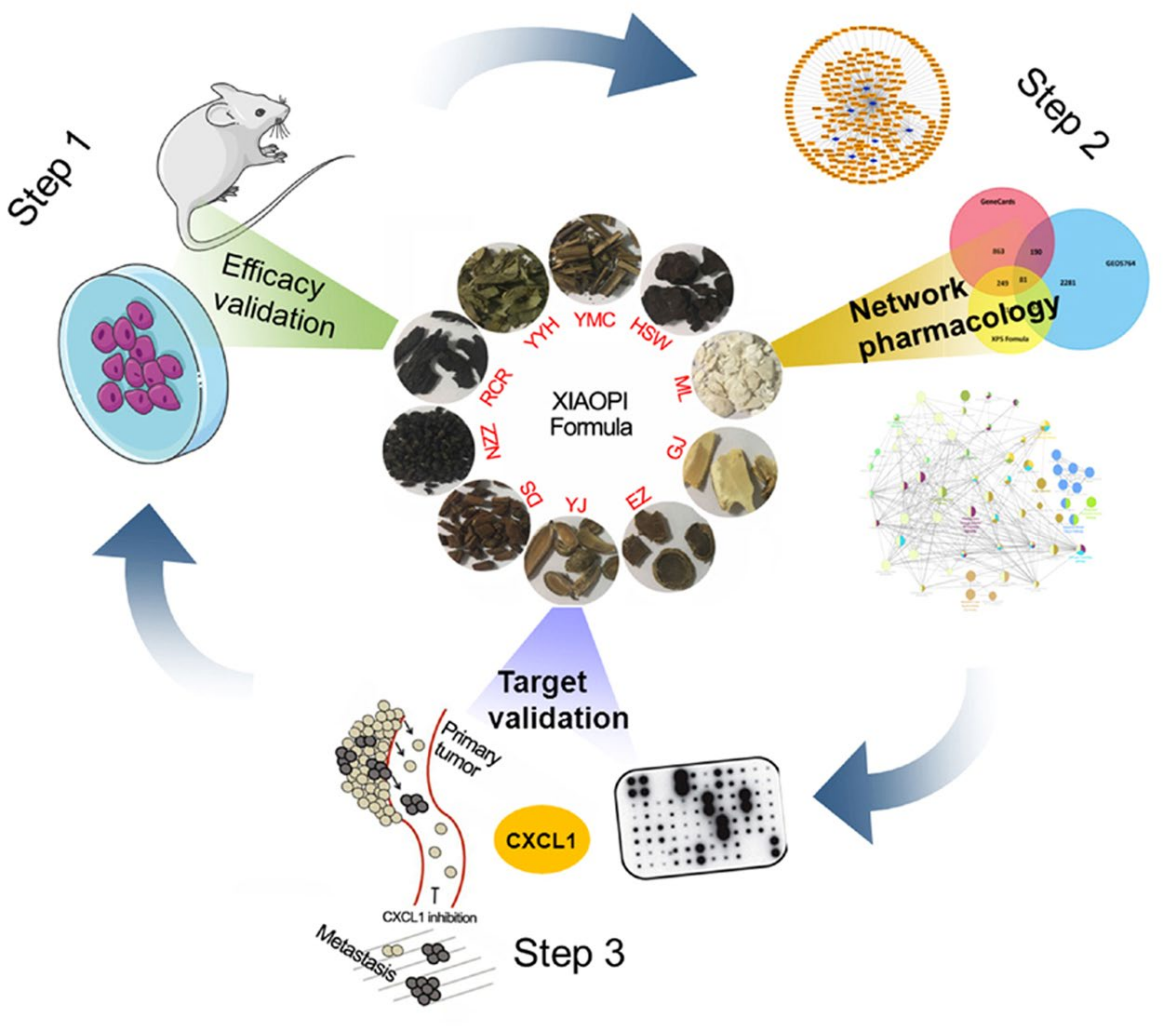
Process overview.

In the present study, we systematically exploited the breast cancer prevention effects and mechanisms of a clinical effective prescription XIAOPI formula. Firstly the breast cancer prevention effects of XIAOPI formula was validated on MMTV-PyMT transgenic mice and *in vitro* cell transformation model. Then the active ingredients in XIAOPI formula were found *via* oral bioavailability and drug-likeness evaluation at a molecular level. By utilizing the active ingredients as bait, we predicted the potential targets and further constructed the drug-target interactions at pharmacological level. We later identified CXCL-1 as the key action gene of XIAOPI formula using cytokine chip analysis. We further validated the importance of CXCL-1 in mediating the anti-cancer effects of XIAOPI formula (Fig. 1). Our work highlights the role of CXCL-1 as a key target in breast cancer development and in the action of XIAOPI formula and also contributes to the exploration of the mechanism underlying TCM and the promotion of its development in the treatment of complex disease.

## Results

### XIAOPI formula inhibits breast cancer tumorigenesis *in vivo*

To confirm the chemopreventive effects of XIAOPI formula, we firstly used a MMTV-PyMT^+/−^ transgenic mouse model to assess the efficacy of XIAOPI formula *in vivo*. The MMTV-PyMT^+/−^ transgenic mice developed spontaneous and luminal-like breast cancer. In particular, mammary hyperplasia can be detected in this model as early as 4 weeks of age, and nearly 100% of mice develop breast cancer by 12–13 weeks, accompanied by the appearance of pulmonary metastasis^22^. For this reason, this model is usually used to evaluate the oncogenic mechanisms underlying breast cancer or the chemopreventive effects of candidate phytochemicals.

**Figure 2:**
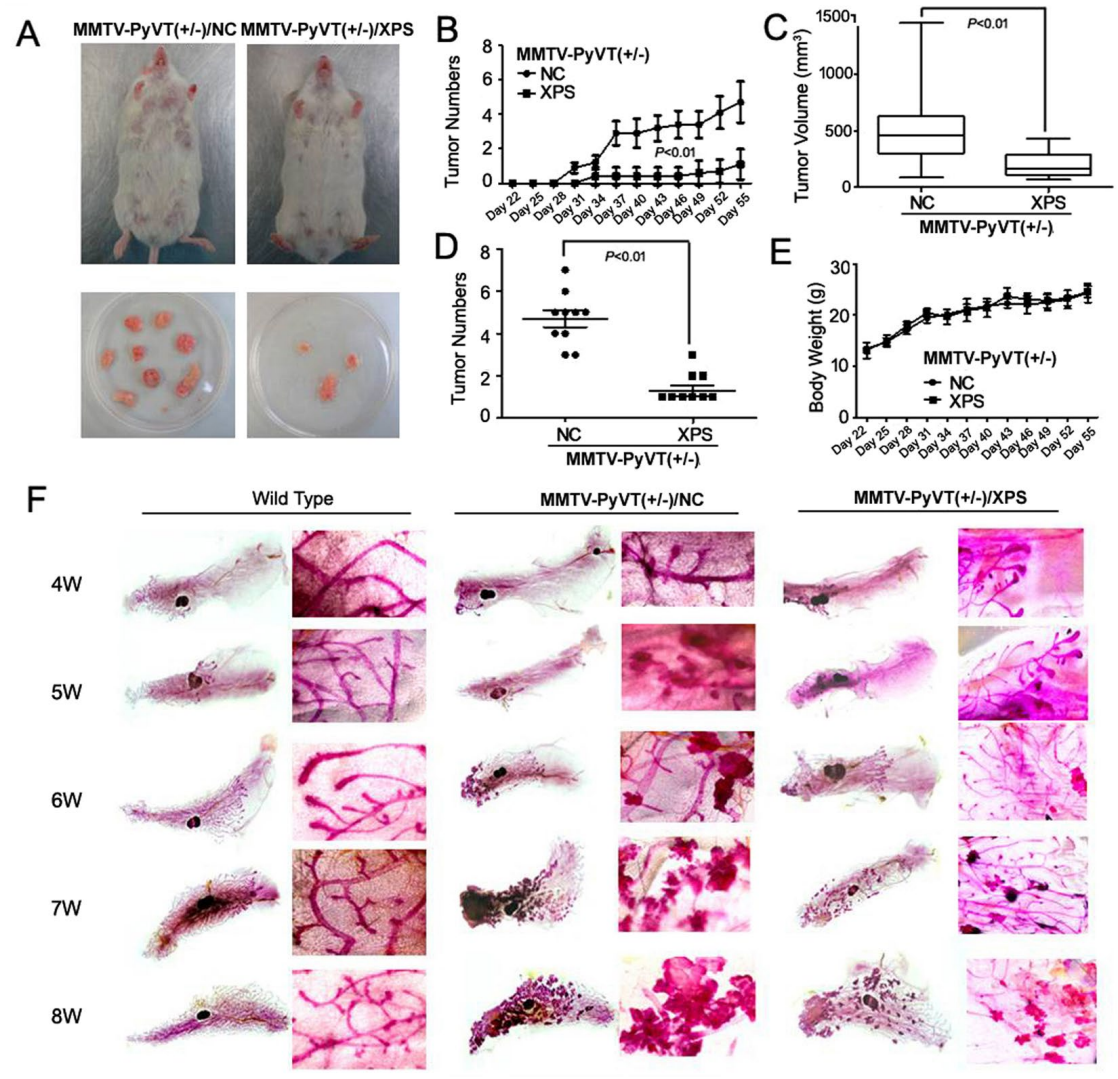
XIAOPI formula delays breast cancer onset *in vivo*. (**A**) Representative mice and tumor in control and XIAOPI groups; XIAOPI formula administration significantly inhibits (**B**) breast cancer onset time, (**C**) tumor volume (mm^3^) and (**D**) tumor number, but brought little influence on (**E**) mice body weight; (**F**) Mammary whole mounting assay revealed that MMTV-PyMT^+/−^ develops malignant lesions as early as the 4^th^ week, while XIAOPI delays malignant transformation to the 5^th^ week.

The cancer preventive activity of XIAOPI formula was assessed by comparing the incidence of palpable lesions at three-day intervals in the transgenic mice in each group. As shown in Fig. 2A, XIAOPI treatment visibly limited the process of mammary oncogenesis. In vehicle controls, tumors appeared as early as the 4^th^ week, while it was delayed one week with XIAOPI intervention (Fig. 2B). Notably, the tumor volume and number in XIAOPI group was significantly inhibited compared with the vehicle group, further confirming the breast cancer suppression effects of the formula (Fig. 2C and D). Meanwhile, XIAOPI brought little influence on mice body weight (Fig. 2E).In order to assess the precise morphological changes during mammary oncogenic process between groups, mammary tissue from the 4^th^ to 8^th^ week was collected from mice in each group and compared by carmine staining analysis. Results showed that the length of ducts, the amount of duct branches, and the number of terminal ends gradually increased with age in wild-type mice. However, in MMTV-PyMT^+/−^ transgenic mice, hyperplastic lesions could be observed as early as the 4^th^ week around duct branches. No lesions were detected in the XIAOPI treatment group until the 5^th^ week. Even at the 8^th^ week, the precancerous lesions were also significantly smaller and fewer in number than in the vehicle control group (Fig. 2F). These findings suggested that XIAOPI formula could block the carcinogenic transformation of mammary tissue efficiently.

### XIAOPI formula inhibits breast cancer lung metastasis and CSCs *in vivo*

**Figure 3:**
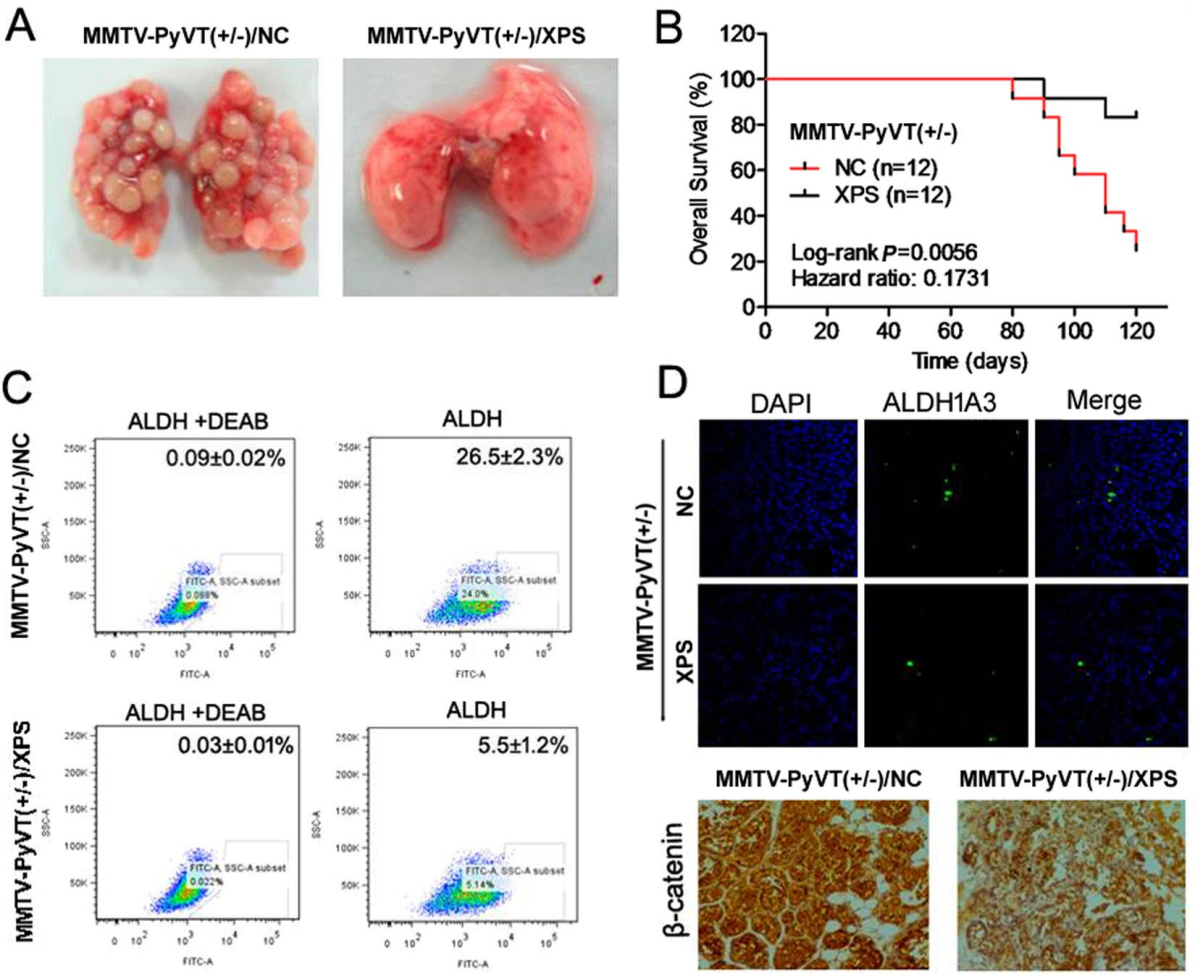
XIAOPI formula inhibits breast cancer lung metastasis *in vivo*. (**A**) Representative picture of lung metastasis lesions in control and XIAOPI groups; (**B**) XIAOPI formula prolongs MMTV-PyMT^+/−^ survival period (*P* = 0.0056, values represented as the mean ± SD, n = 12); (**C**) XIAOPI formula inhibits the CSCs population in the lung metastasis lesion (*P < 0.001, values represented as the mean ± SD, n = 3); (**D**) XIAOPI formula inhibits the number of ALDH^+^ cells and β-catenin expression in the lung section.

Because the MMTV-PyMT^+/−^ transgenic mouse model has a tendency to form metastatic lesions in the lungs, it is rational to determine whether XIAOPI formula can inhibit lung metastasis in breast cancer. As shown in Fig. 3A, at the end of the experimental period, there were fewer metastatic lung nodules in the XIAOPI group than in the vehicle group. The survival curve of XIAOPI-treated mice was significantly longer than in the vehicle control group, which showed serious death events after the 12^th^ week (Fig. 3B). In order to determine whether the decreased lung metastasis events was correlated to smaller numbers of cancer stem cells (CSCs), ALDH assay was carried out to confirm the number of CSCs in the metastatic lesions. As shown in Fig. 3C, as many as 26.5 ± 2.3% CSCs in the vehicle group were positive for ALDH1A3, as opposed to only 5.5 ± 1.2% in the XIAOPI treatment group. Immunofluorescence assay further confirmed that ALDH1A3-postive CSCs were significantly reduced in the XIAOPI-treated group. More importantly, CSCs-related signaling β-catenin expression was also inhibited after long-term XIAOPI administration, further demonstrating that XIAOPI formula might inhibit breast cancer lung metastasis *via* inhibiting CSCs (Fig. 3D).

### XIAOPI formula had little inhibitory effects on breast cancer cells *in vitro*

**Figure 4:**
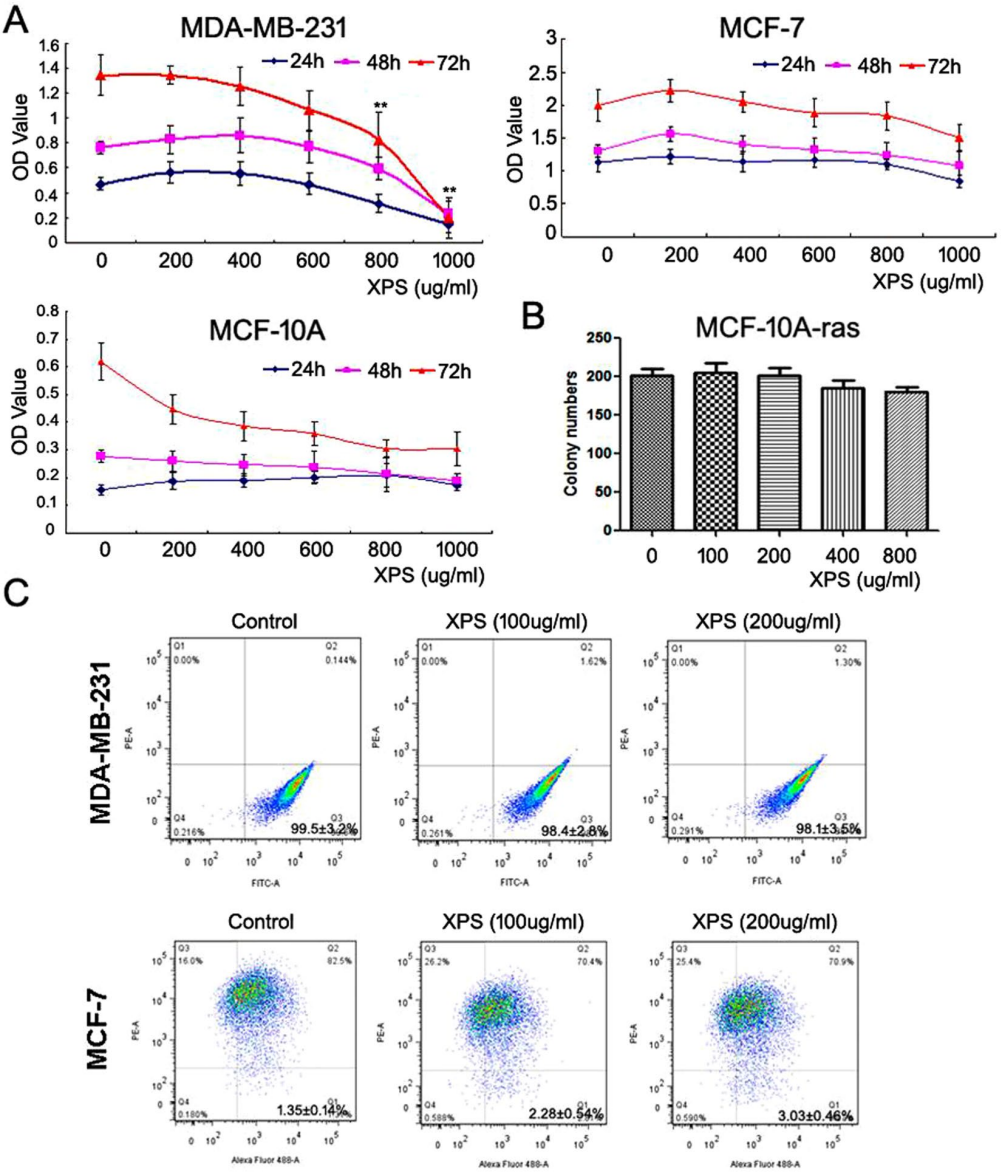
XIAOPI formula brought little influence on breast cancer cell proliferation and CSCs population *in vitro*. (**A**) XIAOPi formula had little proliferation inhibition effects on breast cancer cell line MCF-7 and normal mammary epithelial cell line MCF-10A, but had an obvious toxic effect on MDA-MB-231 on high concentrations from 800 to 1000 μg/ml (**P < 0.01, values represented as the mean ± SD, n = 3); (**B**) XIAOPI formula had little inhibitory effects on the colony growth of *RAS*-transformed MCF-10A cells; (**C**) XP formula had little influence on the subpopulation size of CSCs in both MDA-MB-231 and MCF-7 cells.

Based on the *in vivo* results, it is interesting and important to explore whether XIAOPI had direct inhibitory effects on breast cancer cells. Intriguingly, a cell proliferation assay showed that XIAOPI formula had little inhibitory effect on MCF-7 and MDA-MB-231 breast cancer cells within the range from 0 to 600 μg/ml. At higher concentrations ranging from 600 to 1000 μg/ml, XIAOPI exhibited an inhibitory effect on the proliferation of MDA-MB-231, but it had little effect on MCF-7 cells. XIAOPI had little cytotoxic effect on MCF-10A, a normal mammary epithelial cell line, demonstrating the safety of XIAOPI treatment *in vivo* (Fig. 4A). In order to validate the potential inhibition effects of XIAOPI formula on precancerous cells, the oncogene *RAS* was transformed into MCF-10A cells. These results also confirmed that XIAOPI had little influence on the viability of *RAS*-transformed cells, indicating that the ability of XIAOPI to inhibit breast cancer might not be attributed to the direct toxicity effects on cancer cells (Fig. 4B). Since *in vivo* findings suggested that XIAOPI formula could inhibit breast CSCs, it is necessary to explore whether XIAOPI has similar effects in *in vitro* systems. Flow cytometry assay indicated that XIAOPI formula had little influence on the distribution of CD44 and CD24, the cell surface markers for breast CSCs, indicating that the CSCs-inhibitory effects of XIAOPI formula might be attributed to the tumor microenvironment (Fig. 4C).

### Putative major ingredients and major targets of XIAOPI formula

**Figure 5:**
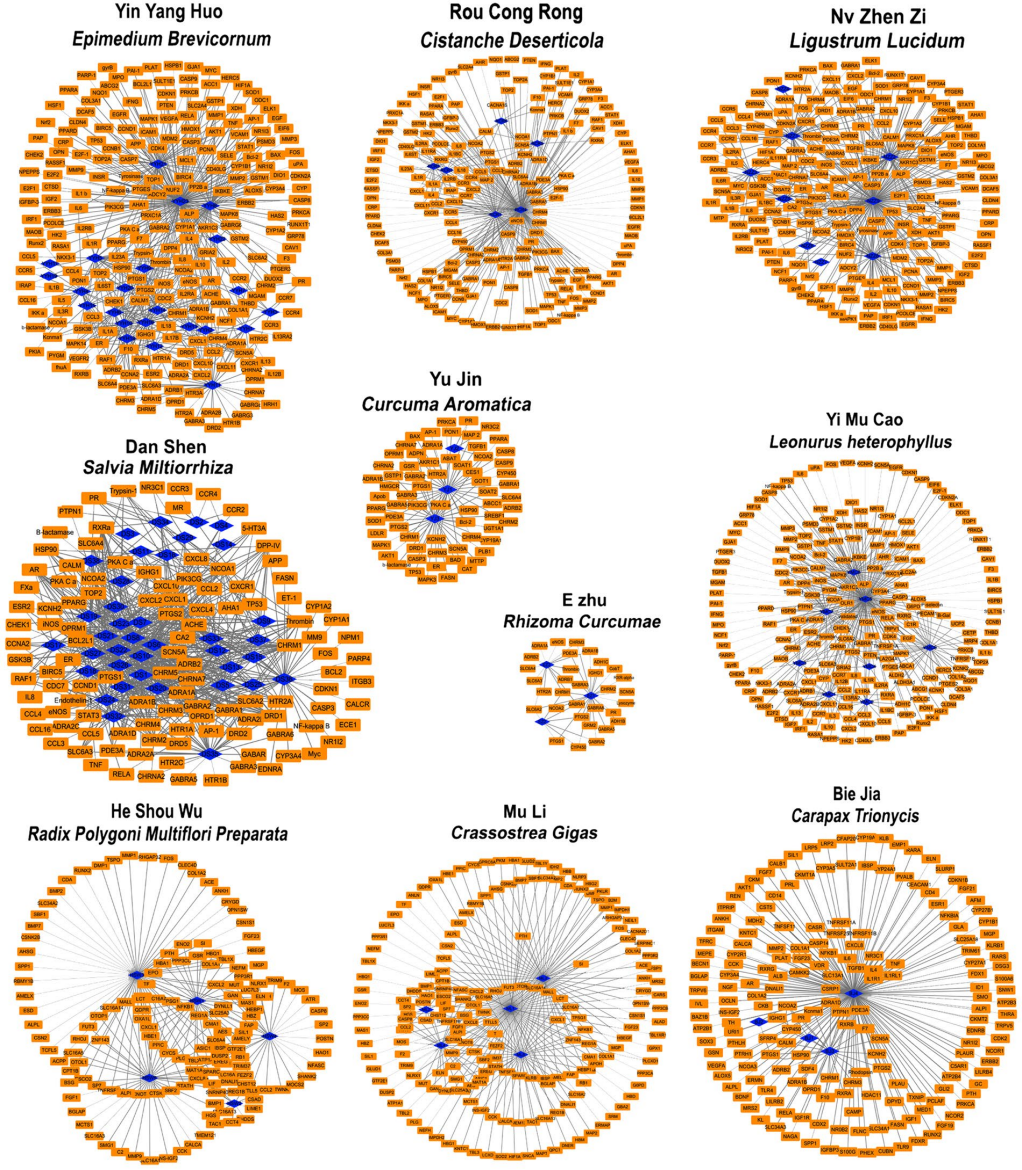
The ingredient-target networks of 10 herbs in XIAOPI formula. The diamond nodes represent ingredients, and the circular nodes represent targets.

**Figure 6:**
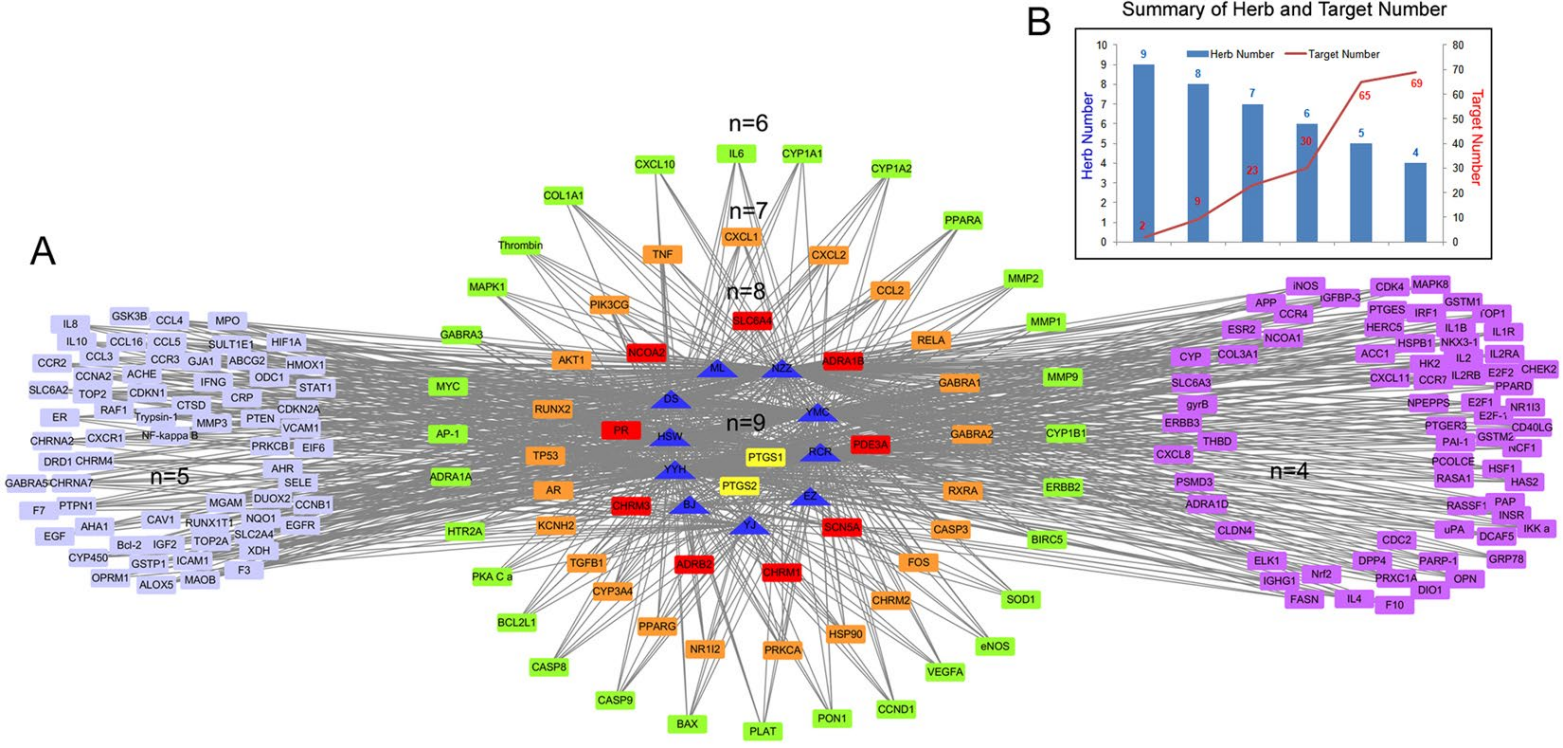
(**A**) The herb-target networks of the 10 herbs. The diamond nodes represent herbs, and the circular nodes represent targets. The targets distributed in a circle represent they are acting by the same number of herbs, which illustrated as “n”; (**B**) the histogram of “Summary of Herb and Target Number”.

Ingredient-target networks were established for all the 10 herbs. The results indicated that the 10 herbs of XIAOPI yielded 105 components and further resulted in 806 potential targets (Fig. 5). Regarding each herb, there were 23 compounds in YYH targeting 246 genes, 6 compounds in RCR targeting 198 genes, 9 compounds in NZZ targeting 215 genes, 38 compounds in DS targeting 115 genes, 3 compounds in YJ targeting 69 genes, 3 compounds in EZ targeting 32 genes, 8 compounds in YMC targeting 223 genes, 6 compounds in HSW targeting 358 genes, 5 compounds in ML targeting 149 genes, and 4 compounds in BJ targeting 182 genes (Fig. 5 and Supplementary Table 1). The common targets for at least 4 herbs are shown in Fig. 6A. Among these genes, 2 were common targets of 9 herbs, 9 were common targets of 8 herbs, 23 were common targets of 7 herbs, 30 were common targets of 6 herbs, 65 were common targets of 5 herbs, and 69 were common targets of 4 herbs (Fig. 6B).

**Figure 7:**
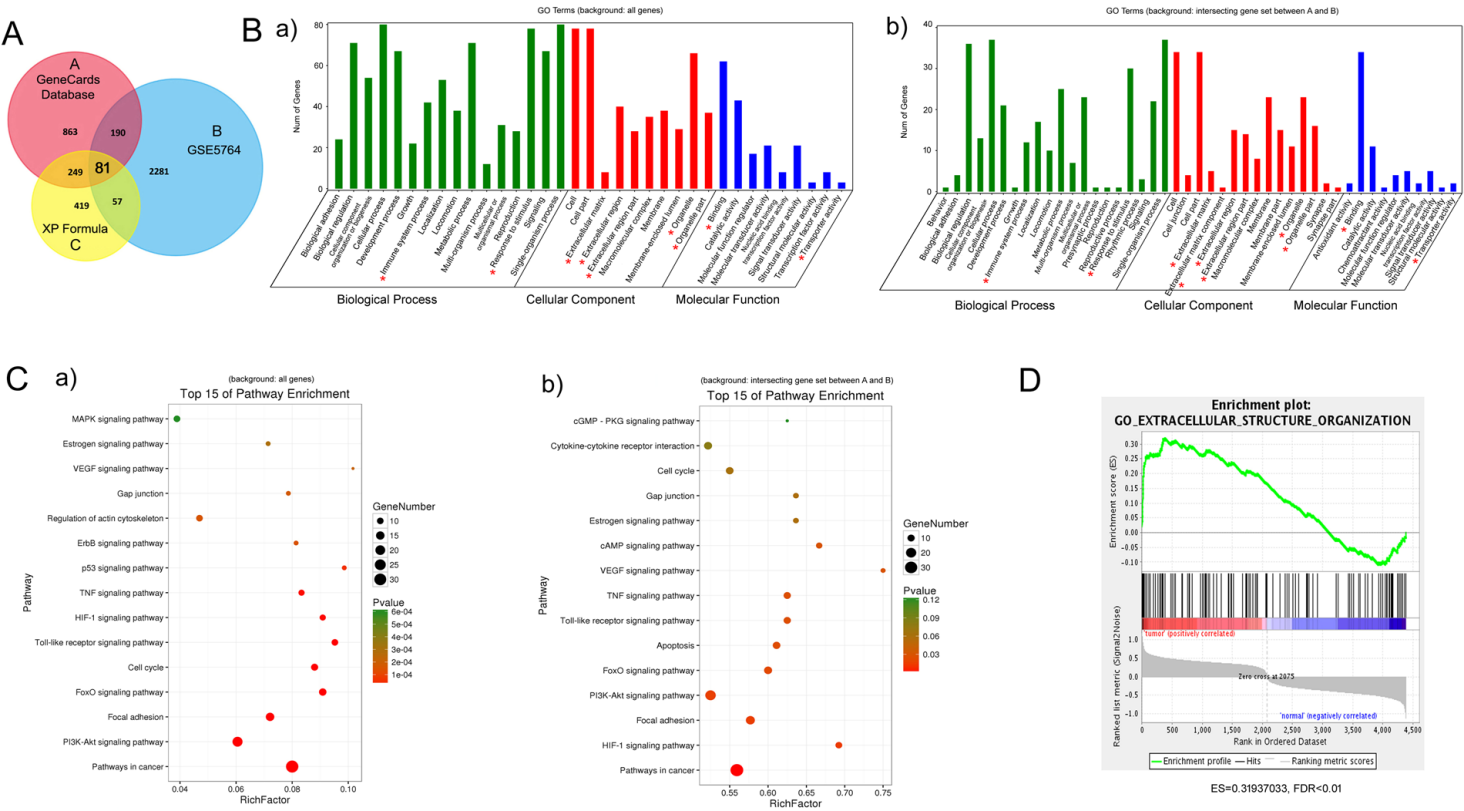
Gene Ontology (GO) and Pathway enrichment analysis of targets in XIAOPI formula. (**A**) Combined with GENECARDS database A and GSE7564 microarray B, it is revealed that a total of 81 genes were closely correlated with anti-breast cancer activities of XIAOPI formula; (**B**) GO terms analysis of the 81 genes containing 3 aspects including molecular function, cellular component and biological process. Either a) all genes background or b) intersecting genes set between A and B background was selected to assess the significance of the 81 gene enrichment, respectively (microenvironment-related functions are labeled with *); (**C**) Pathway enrichment analysis of the 81 genes. Either a) all genes background or b) intersecting genes set between A and B background was selected to assess the significance of the 81 gene enrichment, respectively; (**D**) The representative analysis extracellular structure organization was shown in Fig. 7D (ES = 0.31937033, FDR < 0.01) by GSEA analysis.

**Figure 8:**
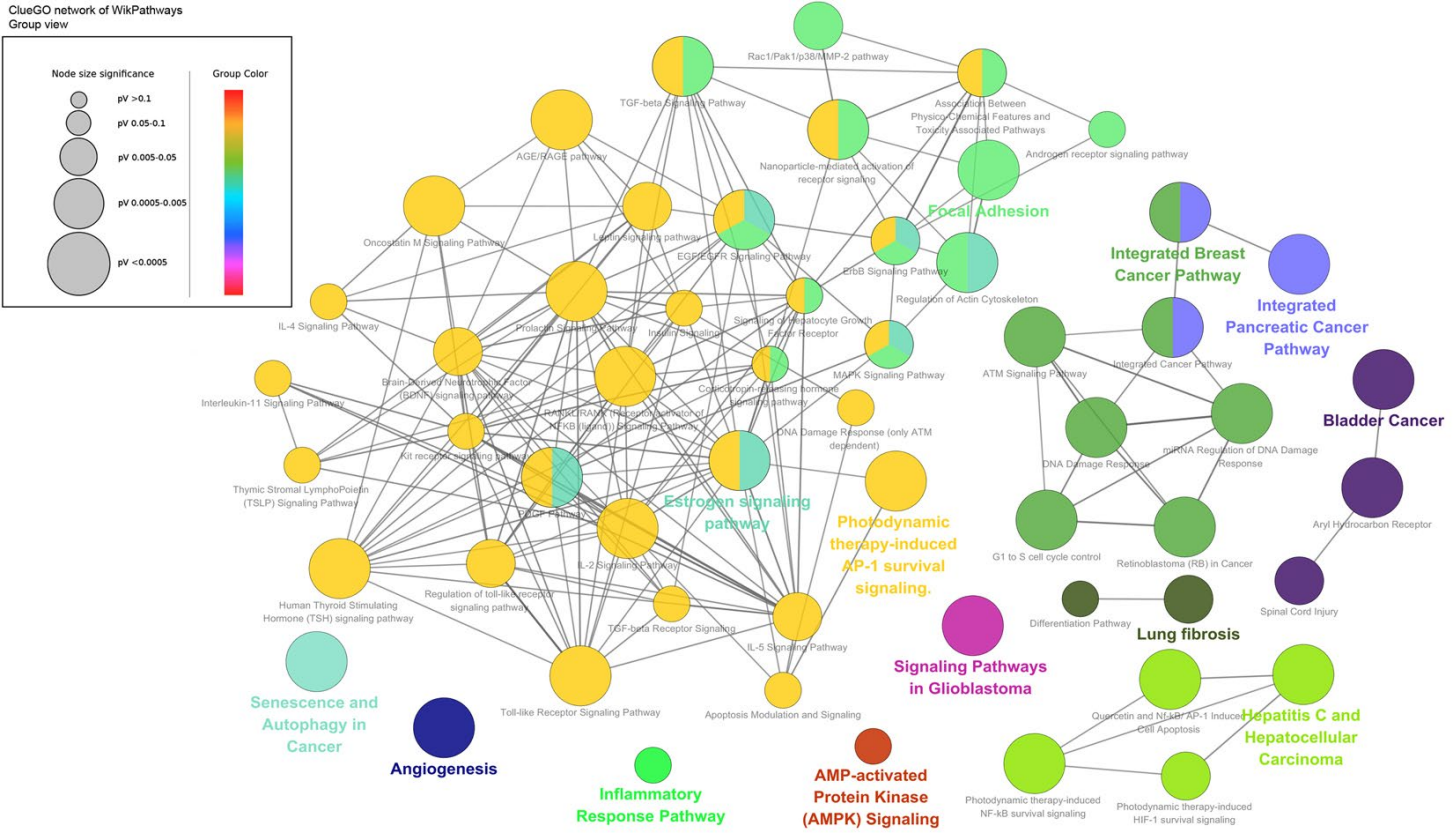
Functional grouped network for the targets of XIAOPI formula by ClueGo WikiPathway analysis (*P* < 0.05). Functional related groups partially overlapped, only the label of the most significant term per group is displayed.

In order to identify the drug-targeting genes influencing breast cancer progression, genes related to breast cancer were also extracted from GENECARDS database and GSE5764 microarray set. As shown in Fig. 7A, there were 1383 breast cancer-associated genes (A, relevance score >5) in the GENECARDS and 2609 validated genes in GSE5764 through GEO2R analysis tool (B, *P* < 0.05). Venn analysis further confirmed that a total of 81 genes were overlapped between the three groups (Supplementary Table 2). For “Gene Ontology (GO) and pathway enrichment analysis”, a proper background gene set was needed to assess the significance of the enrichment. In this study, two random gene sets were picked from either a) all genes or b) intersecting set between A and B as background genes. The purpose of the later is to further investigate whether the enrichment is driven by the targets of XIAOPI components. For GO term analysis (Fig. 7B), Biological Process analysis of the 81 genes revealed that the enrichment on both backgrounds were kept in consistent, except presynaptic process and rhythmic process. Notably, Cellular Component analysis implied that the extracellular matrix region/region part was most significantly associated with XIAOPI action. Further Molecular Function analysis indicated the terms existing in both background include binding, catalytic activity, molecular function/transducer activity, nucleic acid binding transcription factor activity, signal transducer activity, structural molecular activity and transporter activity. For KEGG enrichment analysis (Fig. 7C), the results demonstrated that the interfered pathways of 81 genes co-existing in both backgrounds were mainly responsible for supporting cancer growth and invasion like HIF-1α signaling, focal adhesion signaling, PI3K-Akt signaling, Toll-like receptor signaling, TNF signaling, FoxO signaling, etc. We also performed a gene set (GSE5764 microarray set) enrichment analysis (GSEA) to reveal whether the 81 gene overlap were significantly enriched. 49 of the 81 genes were significantly enriched in the curated gene sets (C2), the enriched pathways were prostate cancer, multi-cancer invasiveness, proliferation, invasive breast cancer and etc. Among them, 34 genes were significantly enriched in breast cancer-related pathways. Besides, 39 of the 81 genes were significantly enriched in GO gene sets (C5), and the enriched pathways were extracellular matrix, extracellular structure organization, epidermis development, exocrine system development and etc. The representative analysis extracellular structure organization was shown in Fig. 7D (ES = 0.31937033, FDR < 0.01). ClueGo WikiPathway analysis (*P* < 0.05) also indicated that multiple cancer-related pathways were significantly involved in the mechanisms of XIAOPI formula, such as focal adhesion, Estrogen signaling pathway, Toll-like receptor signaling, angiogenesis signaling, inflammatory response pathways, and AMPK signaling (Fig. 8).

The tumor microenvironment refers to the cellular environment where the tumor survive, including surrounding blood vessels, immune cells, fibroblasts, bone marrow-derived inflammatory cells, lymphocytes, signaling molecules and the extracellular matrix^23–25^. Interestingly, the definition of TME coincidently met many aspects of XIAOPI-affected signals predicted in above (* indicated in Fig. 7B), we therefore postulated that XIAOPI formula might prevent breast carcinogenesis *via* microenvironment modulation, which would definitely need further experimental validation in the next part.

### Identification of CXCL-1 as the critical target of XIAOPI through cytokine array

**Figure 9:**
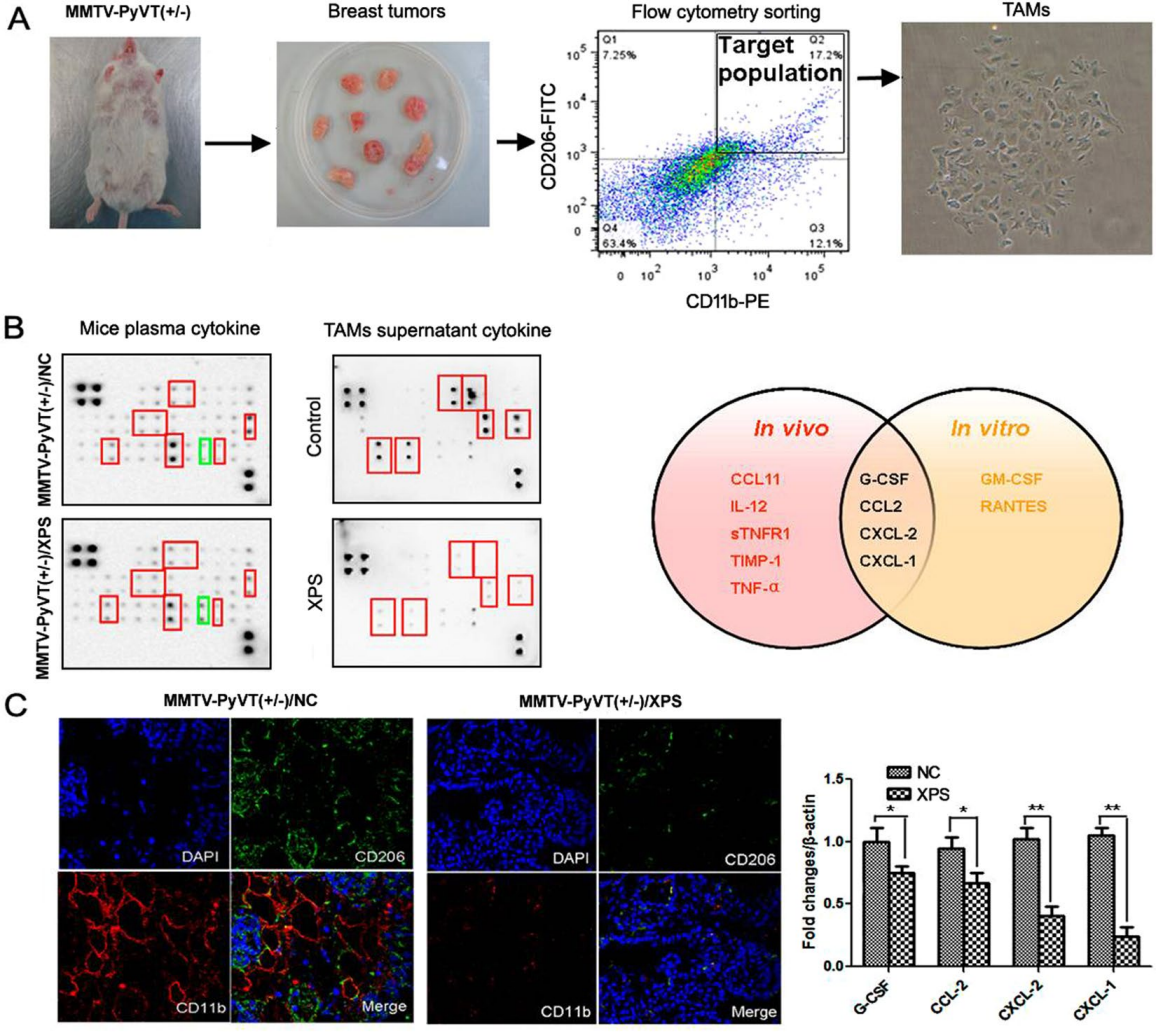
XIAOPI formula targets TAMS/CXCL-1 signaling. (**A**) TAMs were sorted out from tumors of MMTV-PyMT^+/−^ mice by flow cytometry; (**B**) Cytokine array identified G-CSF, CCL2, CXCL-1/2 as common targets of XIAOPI formula between *in vitro* and *in vivo* system; (**C**) Immunofluorescence assay found that the TAMs density was significantly decreased following XIAOPI administration, and qPCR assay further validated that CXCL-1 as the most downregulated gene following XIAOPI administration (*P < 0.05, **P < 0.01, values represented as the mean ± SD, n = 3).

Based on the results of network pharmacology analysis and the critical role of tumor associated macrophages (TAMs) in cancer microenvironment^26^, we sorted out TAMs from the solid tumor of MMTV-PyMT^+/−^ mice using its markers CD11b and CD206 and then treating TAMs with XIAOPI (Fig. 9A). We also collected the plasma from the mice of the vehicle and XIAOPI group. The plasma and TAM supernatants were then subjected to cytokine array analysis. Comparison of the cytokine expression profiles of plasma between control and XIAOPI treatment groups showed there to be 8 kinds of cytokines down-regulated following XIAOPI administration including CCL11, G-CSF, IL-12p40/70, IL-12/p70, CCL2, CXCL-2, sTMFR1 and TNF-α, while the expression of TIMP-1, a cancer metastasis inhibition gene, was upregulated by XIAOPI. Regarding TAMs supernatants, 6 kinds of cytokines were found downregulated after XIAOPI administration, including GM-CSF, G-CSF, CCL-2, CXCL-2, CXCL-1 and RANTES. By intersecting the cytokine change profiles between *in vivo* and *in vtiro*, we found that a total of 4 cytokines (G-CSF, CCL2, CXCL-2, CXCL-1) share the similar changes response to XIAOPI treatment (Fig. 9B). Immunofluorescence assay further validated decreased intensity of TAMs in the tumors of XIAOPI group, and qPCR demonstrated that all 4 of cytokines were downregulated by XIAOPI treatment and CXCL-1 showed the most pronounced reduction (Fig. 9C).

### XIAOPI formula inhibits breast cancer metastasis *via* downregulating TAM-secreted CXCL-1

**Figure 10:**
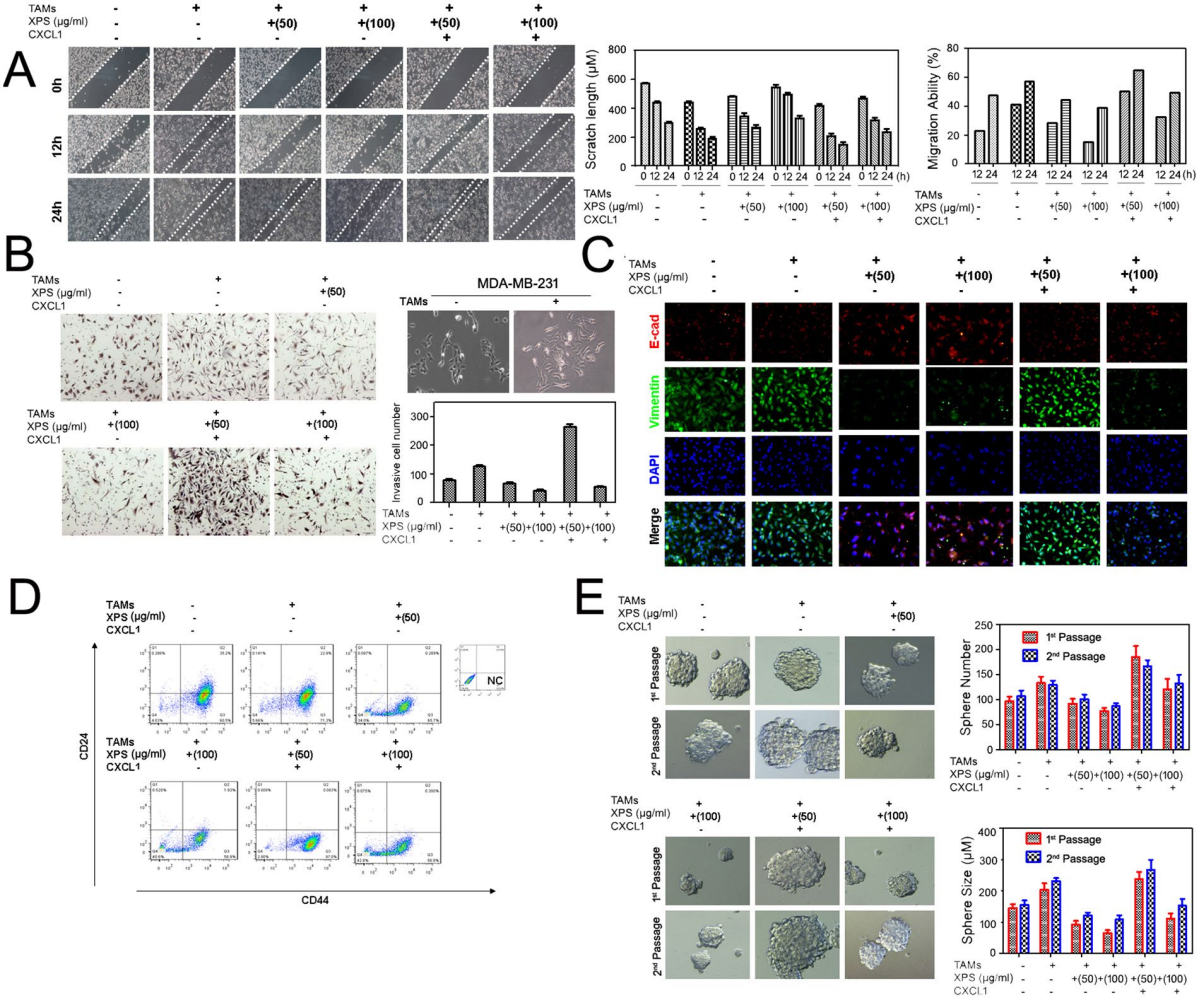
XIAOPI formula inhibits breast cancer metastasis *via* CXCL-1. (**A**) Wound healing assay indicated that XIAOPI formula could inhibit MDA-MB-231 migration dose-dependently, but CXCL-1 treatment inversely counteracted against XIAOPI’s effects; (**B**) Transwell assay demonstrated that XIAOPI formula could inhibit MDA-MB-231 cell invasion ability, and CXCL-1 administration restored the formula’s inhibition effects; (**C**) Immunofluorescence assay validated that XIAOPI formula could activate the EMT process, presenting as increased expression of E-cadherin and decreased level of vimentin, but CXCL-1 overexpression reverse the EMT process; (**D**) Flow cytometry assay also demonstrated that XIAOPI formula could inhibit the CSCs subpopulation, but CXCL1 administration in the TAMs supernatants counteracted with XIAOPI formula; (**E**) Mammosphere formation assay indicated that the size and number of mammospheres were both inhibited by XIAOPI administration, but re-increased following CXCL-1 treatment (*P < 0.05, **P < 0.01, all values represented as the mean ± SD, n = 3).

In order to validate the critical role of CXCL-1 in mediating the anti-cancer activities of XIAOPI formula, we carried out *in vitro* verification experiments using MDA-MB-231 cells. Wound healing and transwell assay showed that the supernatants of TAMs could stimulate cell migration and metastasis over vehicle-treated cells. However, XIAOPI formula inhibited the TAM-induced cell migration in a dose-dependent manner. Intriguingly, CXCL-1 administration was found to aggravate the migration stimulating effect of TAMs. CXCL-1 abolished the ability of XIAOPI formula to inhibit metastasis, indicating that CXCL-1 is a critical mediator responsible for the anti-cancer activities of the formula (Fig. 10A and B). Immunofluorescence assay also demonstrated that TAM supernatants stimulated the EMT process as evidenced by increased expression vimentin and decreased level of E-cadherin. By contrast, XIAOPI formula was found to efficiently inhibit EMT process induced by TAMs. What’s more important, CXCL-1 was also found to promote the EMT transformation and relieve the bioactivity of XIAOPI formula (Fig. 10C). These findings not only indicated that the metastasis-promoting effects of CXCL-1 were closely associated with EMT mechanism but that the anti-metastasis effects of XIAOPI formula might be mediated through inhibition of the EMT process. Because the EMT process is closely associated with the functions of CSCs, we therefore further evaluated its influence on the functions of CSCs. Flow cytometry analysis revealed that TAM supernatants could increase the population of CSCs, and this effect was strengthened by addition of CXCL-1. However, XIAOPI formula was also found to inhibit the increase in the CSC population after TAM treatment. Similarly, CXCL-1 was found to weaken the bioactivity of XIAOPI formula, which was consistent with our findings given above (Fig. 10D). Mammosphere assay also validated that CXCL-1 could assist TAM supernatants to increase the size and number of mammosperes, while XIAOPI formula inhibited their effects (Fig. 10E). All these findings demonstrated the critical role of CXCL-1 in mediating the cancer prevention activities of XIAOPI formula and highlighted the significant effects of CXCL-1 in promoting breast cancer metastasis.

## Discussion

Cancer is a heterogeneous disease. Its initiation is correlated not only with oncogene activation and tumor suppressive gene inactivation, but also closely associated with aberrant immune functions, stem cells disorder and cytokine disregulation^27^. With regard to breast cancer, BRCA1/2 mutations are reported to be mainly responsible for the development of hereditary breast cancer, and corresponding gene screening has become a routine tool for decreasing the risk ratio of mammary malignancies. However, increasing amounts of evidence have indicated that multiple factors also contribute to breast cancer development, such as estrogen level, estrogen receptor status, inflammation, macrophage activity, DNA repair ability, and overactivation of mTOR survival signaling^28,29^. The breast cancer molecular phenotype also undergoes dynamic changes, such as a transition of subtype from HER2-negative to -positive, increased cancer stem cell population, and lymphocyte infiltration^30^. For this reason, breast cancer therapy has been developed into a multidisciplinary treatment including surgery, chemo-/radiotherapy, endocrine therapy and targeted treatment. There is growing awareness that single-target blocking strategies cannot eliminate cancer cells, since there exists compensatory response in the signaling network. In this way, a cocktail strategy or multi-targeting protocol has drawn much more attention nowadays^31^. Coincidently, TCM is appreciated for its multi-compounds, multi-targets and multi-pathways. TCM has exhibited its mystic and profound therapeutic effects on some incurable diseases including autoimmune disorders, chronic inflammation and cancer.

In the present study, we demonstrated a clinical prescription XIAOPI formula could efficiently prevent breast cancer progression in MMTV-PyMT^+/−^ xenografts. However, it did not show a direct inhibition effects on breast cancer cells or *RAS*-transformed mammary epithelial cells, indicating that the anti-cancer activities of XIAOPI formula might be attributed to systemic regulation, especially for the tumor microenvironment. Because the XIAOPI formula consists of 10 herbs, which might contain thousands of phytochemicals and molecular targets, current techniques might be difficult to precisely explain its underlying mechanisms. It is of great interest to elucidate the molecular network of XIOPI formula using a systemic-based strategy. Network pharmacology is recently emerging as a powerful tool for understanding the complex action mechanisms of TCM formulas. Through database searching and network analysis, a total of 105 active compounds and 806 genes were finally identified in the XIAOPI formula. Further gene crosstalk analysis identified 81 genes were closely correlated to breast cancer development. Through Gene Ontology and Pathway enrichment analysis, the 81 genes were validated to participate in various biological processes during cancer initiation, such as response to stimulus, immune system response, extracellular signals, organelle, binding activity, as well as focal adhesion signaling, etc. ClueGo WikiPathway analysis revealed that most signaling were belonged to focal adhesion, Toll-like receptor signaling, angiogenesis signaling and inflammatory response pathways, further indicating that XIAOPI formula might act through tumor microenvironment. Cytokine array further indicated that multiple cytokines were affected by administration of XIAOPI formula both *in vitro* and *in vivo*. Among the 32 kinds of cytokines detected, CXCL-1 has been shown to be the cytokine most profoundly affected by the formula. Other studies also reported the application of bioinformatics analysis and network pharmacology in breast cancer. Cong Fu *et al*. established a literature-mined human signaling network by integrating data on ubiquitin-mediated protein half-lives. Their study demonstrated that the 26 S proteasome genes were significantly correlated with breast cancer tumor progression and metastasis, and predicted clinical outcome of breast cancer patients^32^. Zaman N *et al*. predicted breast cancer subtype-specific drug targets based on signaling network assessment of mutations and copy number variations^33^. McGee SR *et al*. revealed a positive regulatory loop in the PIK3CA-mutated breast cancer predicting survival outcome based on Cancer Hallmark Network Framework^34^. Taken together, our work has great implications for the development of breast cancer prognostic markers by integrating bioinformatics and network pharmacology followed by experimental validation.

CXCL-1 is a member of CXC the chemokine family, and it was firstly reported to support melanoma growth by Richmond *et al*.^35^. Recent progress also indicated the critical role of CXCL-1 in multiple malignancies. CXCL-1 high expression in breast cancer cells could attract CD11b^+^Gr1^+^ myeloid cells into the tumor and therefore resulting in chemoresistance and metastasis^36^. CXCL-1 overexpression was also found to be significantly closely with gastric cancer progression and poor survival. CXCL-1 depletion was capable of inhibiting the migration and invasion ability of gastric cancer cells^37^. Similar findings were also confirmed in bladder cancer. CXCL-1 levels have been shown to be significantly higher in invasive bladder cancer and to promote metastasis *via* regulating MMP-13 expression^38^. Notably, CXCL-1 was found to be secreted from tumor stromal cells and affect cancer progression *via* a paracrine signaling. CXCL-1 secreted from lymphatic endothelial cells could promote gastric cancer migration, invasion and adhesion abilities *via* activating integrin β1/FAK/AKT signaling^39^. CXCL-1 was also found to be a cytokine secreted by tumor-associated macrophage, which recruits myeloid-derived suppressor cells to form pre-metastatic niche and led to liver metastasis from colorectal cancer^40^. The current study demonstrated that after administration of XIAOPI formula, the density of TAMs decreased significantly and the level of CXCL-1 was also inhibited in both mouse plasma and cellular supernatants. When CXCL-1 cytokine was co-administrated with XIAOPI formula, the anti-metastatic property of XIAOPI formula was blocked, indicating that CXCL-1 might be principal gene involved in the network regulating the action of XIAOPI formula.

Taken together, our study provides a candidate formula for preventing breast cancer growth and metastasis, and highlights the role of CXCL-1 in mediating the bioactivities of XIAOPI formula. However, further research is still needed to clarify the pathological significance of CXCL-1 in promoting breast cancer metastasis and to identify the potential CXCL-1 inhibitors from the formula *via* high-throughput screening technique.

## Materials and Methods

### Preparation of XIAOPI Formula

XIAOPI formula consists of 10 herbs including Epimedium Brevicornum (Chinese name Yin Yang Huo, YYH), Cistanche Deserticola (Chinese name Rou Cong Rong, RCR), Leonurus Heterophyllus (Chinese name Yi Mu Cao, YMC), Salvia Miltiorrhiza (Chinese name Dan Shen, DS), Curcuma Aromatica (Chinese name Yu Jin, YJ), Rhizoma Curcumae (Chinese name E Zhu, EZ), Ligustrum Lucidum (Chinese name Nv Zhen Zi, NZZ), Radix Polygoni Multiflori Preparata (Chinese name He Shou Wu, HSW), Crassostrea Gigas (Chinese name Mu Li, ML)and Carapax Trionycis (Chinese name Bie Jia, BJ).The mixture was treated by ultrasound for 1 h followed by heating twice at 100 °C for 30 min each. The supernatant was concentrated by rotary evaporation and stored overnight at −80 °C. The freezing supernatant was then treated with a freeze dryer for 48 h to find the raw aqueous extract powder. The production ratio was 14.6–15.2%. HPLC analysis of XIAOPI aqueous extract was conducted on an Ultimate AQ-C18 column (250 mm × 4.6 mm, 5 μm). The mobile phase consisted of (A) acetonitrile and (B) water using a gradient elution of 5–20% A at 0–40 min, 20–30% A at 40–50 min and 30–95% A at 50–80 min. The solvent flowrate was 1.0 mL/min and the column temperature was ambient. The results showed that the chemical fingerprints at 200 nm were consistent across different batches (Supplementary Figure 1). The powder was dissolved in phosphate buffer solution and passed through 0.45 μm filter for later use.

### Breast cancer xenografts and mammary whole mounting assay

All animal studies involving animal experiments were reviewed and approved by the University of Hong Kong’s Committee for Ethical Review of Research. All experiments were performed in accordance with the guidelines and regulations issued by the Administration Office of Laboratory Animals at Hong Kong. MMTV-PyMT^+/−^ transgenic mice, a spontaneous breast cancer generation model, was used to confirm the cancer prevention effects of XIAOPI formula. Animals were divided into three groups including the wild type (WT) group, MMTV-PyMT^+/−^ group, and MMTV-PyMT^+/−^ mice treated with XIAOPI group. XIAOPI formula was administrated to MMTV-PyMT^+/−^ mice at 0.5 g/kg/d since the 4^th^ week after birth by oral gavage. The number of tumors, time of tumor appearance, tumor volume, and body weight were recorded at three-day intervals. Tumor volume was calculated using the following formula: volume (mm^3^) = width^2^ × length/2. Tumor tissues were removed at the end of the experiment and subjected to histological examination or flow cytometry analysis. The lung metastasis nodules were calculated and compared between groups. The mammary glands of PYMT mice were excised, and whole-mounts stained with carmine alum were analyzed as described previously^41^. In particular, the fourth abdominal mammary gland was excised during necropsy, spread on glass slides for 10 min, and fixed in Carnoy’s fixative (6 parts 100% ethanol, 3 parts chloroform, and 1 part glacial acetic acid) for 4 h. Subsequently, the tissue was washed in 70% ethanol for 15 min, and the ethanol was changed gradually to distilled water, with a final rinse in distilled water for 5 min. Staining was carried out overnight in carmine alum. The tissue was then dehydrated in graded alcohol solutions (70, 95, and 100% for 30 min each) and cleared in two changes of xylene (30 min each), mounted, and cover slipped using Permount. Whole mounts were recorded using a SPOT FLEX® color digital camera (Diagnostic Instruments, Inc. Sterling Heights, MI, U.S.).

### Cell culture

The human breast cancer cell lines MDA-MB-231 and MCF-7 were obtained from the American Type Culture Collection. The cells were cultivated in medium (L-15 for MDA-MB-231; 1640 for MCF-7) supplemented with 10% FBS and 1% penicillin and streptomycin (Gibco Life Technologies, Lofer, Austria) at 37 °C in a humidified incubator with or without 5% CO2. The normal mammary epithelial cells MCF-10A were cultured in DMEM/F12 supplemented with 5% horse serum, 1% penicillin and streptomycin, 20 ng/ml recombinant human epidermal growth factor (EGF), 0.5 μg/ml hydrocortisone, 100 ng/ml cholera toxin and 10 μg/ml insulin. The sorted MDA-MB-231 cancer stem cells (CSCs) were subjected to *in vitro* propagation in DMEM/F12 medium supplemented with 1% penicillin and streptomycin, B27 (Invitrogen, Carlsbad, CA, U.S.), 20 ng/ml hEGF (BD Bioscience, Bedford, MA, U.S.), 5 μg/ml insulin and 0.4% BSA for the molecular mechanism study.

### Flow cytometry analysis

Primary mouse mammary cells or breast cancer cell lines (MDA-MB-231 and MCF-7) were washed with PBS and then harvested with trypsin. The detached cells were washed with PBS containing 1% FBS (wash buffer), and resuspended in the wash buffer (10^6^ cells/100 μl). For ALDEFLUOR assay, the experiment was performed using aldehyde dehydrogenase-based cell detection kit (Stem Cell Technologies, Grenoble, France) as described previously. Briefly, 2 × 10^5^ cells were suspended in Aldefluor® assay buffer containing ALDH substrate (Bodipy-Aminoacetaldehyde) and incubated for 45 min at 37 °C. As a reference control, the cells were suspended in buffer containing Aldefluor® substrate in the presence of diethylaminobenzaldehyde (DEAB), a specific ALDH1 enzyme inhibitor. The brightly fluorescent ALDH1-expressing cells (ALDH1high) were detected by a 488 nm blue laser. For the *in vitro* stem cell analysis/sorting, cells were incubated with combinations of fluorescence-conjugated monoclonal antibodies obtained from BD Biosciences (San Diego, CA, U.S.) against human CD44-FITC and CD24-PE at 4 °C in the dark for 40 min and then washed once with PBS. FITC- and PE-labeled isotype IgG1 served as the negative control.

### Immunofluorescence assay

Tumor samples obtained from *in vivo* studies were fixed in 4% paraformaldehyde, dehydrated in 70% ethanol, paraffin embedded, and sectioned (4 μm). For immunofluorescence analysis, paraffin-embedded tumor sample sections were de-paraffinized in xylene twice for 10 min each and rehydrated using a graded series of ethanol. The sections and 4% paraformaldehyde fixed cells were permeabilized with 0.2% triton X-100. After blocking in 10% goat serum for 1 h, the slide was incubated with primary antibodies including CD206, CD11b, ALDH1A3, E-cadherin and vimentin (ABclonal, Cambridge, MA, U.S.) overnight at 4 °C, followed by secondary fluorescence-labeled antibodies (Santa Cruz, CA, U.S.) for 2 h at room temperature. Finally, the samples were incubated with DAPI for nuclear staining and the signal was detected using a confocal microscope.

### Herb-ingredient-target interaction analysis

The chemical ingredients were collected from TCM databases, including the Traditional Chinese Medicine Systems Pharmacology (TCMSP) Database (http://lsp.nwsuaf.edu.cn/tcmsp.php), the Traditional Chinese Medicine Integrated Database (TCMID, http://www.megabionet.org/tcmid/) and the BATMAN-TCM (http://bionet.ncpsb.org/batman-tcm/). The ingredients were screened according to drug-likeness (DL) and oral bioavailability (OB) values, and the ingredients were retained if DL ≥ 0.18 and OB ≥ 30, a criterion suggested by TCMSP database^42^. Specifically, the ingredients of ML were inorganic compounds with low DL values; therefore they were not screened by these criteria. The ingredient-target networks were constructed for these herbs using Cytoscape software (Version 3.2.1).

### Gene Ontology and Pathway enrichment analysis

The breast cancer-associated targets were collected from GeneCards database (http://www.genecards.org/). The dataset GSE5764, based on the platform of Affymetrix GeneChip^®^ Human Genome U133 Plus 2.0 Array, was retrieved from the National Center for Biotechnology Information (NCBI) Gene Expression Omnibus (GEO) database (http://www.ncbi.nlm.nih.gov/geo) and then analyzed by GEO2R (http://www.ncbi.nlm.nih.gov/geo/geo2r/). The differentially expressed genes (DEGs) were submitted to the Database for Annotation, Visualization and Integrated Discovery (DAVID; http://david.abcc.ncifcrf.gov/). The significant enrichment analysis of DEGs was assessed based on the gene ontology (GO) and Kyoto Encyclopedia of Genes and Genomes (KEGG; http://www.genome.jp/kegg/kegg2.html). For Gene Ontology (GO) analysis, it fell into 3 categories: molecular function, cellular component and biological process. The basic unit of GO was GO-term. Each GO-term belonged to a type of ontology. GO enrichment analysis provided all GO terms that significantly enriched in DEGs comparing to the genome background, and filtered the DEGs that corresponded to biological functions. Firstly, all DEGs were mapped to GO terms in the Gene Ontology database (http://www.geneontology.org/), gene numbers were calculated for every term, significantly enriched GO terms in DEGs comparing to the genome background were defined by hypergeometric test. The calculating formula of *P*-value was:

$$P=1-\sum _{i=0}^{m-1}\frac{(\begin{array}{c}M\\ {\rm{i}}\end{array})\,(\begin{array}{c}N-M\\ n-i\end{array})}{(\begin{array}{c}N\\ n\end{array})}$$

Here N was the number of all genes with GO annotation; n was the number of DEGs in N; M was the number of all genes that were annotated to the certain GO terms; m was the number of DEGs in M. The calculated *P*-value were gone through the false discovery rate (FDR) Correction, taking FDR ≤ 0.05 as a threshold. GO terms meeting this condition were defined as significantly enriched GO terms in DEGs. This analysis was able to recognize the main biological functions that DEGs exercised. For KEGG Pathway enrichment analysis, the calculating formula was the same as that in GO analysis. For Gene Set Enrichment Analysis (GSEA) analysis, 3 major steps included calculation of an enrichment score (ES), estimation of significance level of ES, and adjustment for multiple hypothesis testing by FDR calculating based on GSEA java software^43^.

### Wound-healing and transwell migration assay

For wound healing assay, 2*10^5^ cells were seeded on a 24-well plate. When they grew to full confluence, a ‘wound’ was made in the middle of a culture plate with a 10 μl pipette tip for MDA-MB-231. The wound-healing process was recorded at 0 h, 12 h or 24 h after the scratch under a 10 × objective microscope. The wound healing rate was quantified as the distance of wound recovered versus that of the original wound. With regard to transwell assay, transwell chambers (8 μm, Milipore) were used for cell invasion. The bottom chamber was filled with culture medium containing 10% FBS. 1*10^5^ cells were suspended in serum-free medium and plated in the upper chamber with or without drug treatment. After incubation for 24 h, the cells were removed from the upper-chamber using a cotton swab. Cells penetrated and attached to the bottom of the filter were fixed with 4% formaldehyde in PBS, followed by 20 min staining of 0.5% crystal violet and then subjected to imaging under a 20 × objective. Statistical analysis of the number of invading cells was performed in three independent experiments and results were averaged from five image fields.

### Cytokine array analysis

Mouse cytokine antibody array C2 kits were purchased from RayBiotech (Norcross, GA, U.S.). The cytokine layout is listed in Supplementary Figure 2. Briefly, cell supernatants from TAMs or mice plasma were collected before and after BM treatment. After antibody array membranes were blocked in 5% BSA for 30 min at room temperature, cell supernatants were cultured with antibody arrays overnight at 4 °C and washed for three times. Biotinylated antibody cocktail was then incubated with the membranes for 2 h, followed by signal amplification with HRP-streptavidin. Finally, the signals were detected by chemiluminescence method with ECL kit purchased from GE Healthcare (Buckinghamshire, U.K.).

### Real-time PCR analysis

TRIzol reagent (Invitrogen, Carlsbad, CA, U.S.) was used to extract total RNA, followed by reverse transcription reaction using the first-strand cDNA synthesis kit (Roche, Mannheim, Germany). A SYBR Green Kit (Roche, Mannheim, Germany) was used to perform real-time PCR analysis on a Roche LightCycler 480 detector. PCR reaction conditions were set as follows: 95 °C for 10 min followed by 40 cycles of 95 °C for 10 s, 55 °C for 30 s, and 72 °C for 1 min. The target gene expression was calculated using 2^−ΔΔ^Ct and normalized to the housekeeping gene. The primers’ sequences were as follows: G-CSF (F, gctgctggagcagttgtg; R, gggatccccagagagtgg); CCL-2 (F, catccacgtgttggctca; R, gatcatcttgctggtgaatgagt); CXCL-2 (F, aaaatcatccaaaagatactgaacaa; R, ctttggttcttccgttgagg); CXCL-1 (F, gactccagccacact ccaac; R, tgacagcgcagctcattg); GAPDH (F, aagagggatgctgccctta; R, ttgtctacgggacga ggaaa).

### Statistical analysis

Data analysis was performed with Statistical Product and Service Solutions (SPSS) 19.0 software. The data are expressed as mean ± SD. The student’s t-test was used to compare the statistically significant difference between groups. The significance of multiple groups was compared using one-way analysis of variance (ANOVA) followed by the Dunnett’s post hoc test. A value of *P* < 0.05 was considered significant.

## Electronic supplementary material

Supplementary files
