# Supplementary material for: Network-pharmacology-based validation of TAMS/CXCL-1 as key mediator of XIAOPI formula preventing breast cancer development and metastasis
Source: Sci Rep. 2017 Nov 6;7:14513. doi: 10.1038/s41598-017-15030-3 (PMC5674025; doi:10.1038/s41598-017-15030-3)
Supplement: Supplementary file 1 — Supplementary files [file 41598_2017_15030_MOESM1_ESM.pdf]

Network-pharmacology-based validation of TAMS/CXCL-1 as key mediator of XIAOPI formula preventing breast cancer development and metastasis

Neng Wang[1, 2], Yifeng Zheng[1, 2, 3], Jiangyong Gu[1], Youli Cai[1, 2], Shengqi Wang[1, 2, 3], Fengxue Zhang[2], Jianping Chen[4], Honglin Situ[1, 2], Yi Lin[1, 2], Zhiyu Wang[1, 2, 3*]

[1]The Research Centre for Integrative Medicine, Guangdong Provincial Academy of Chinese Medical Sciences, Guangzhou University of Chinese Medicine, Guangzhou, Guangdong, China
[2] Integrative Research Labrotary of Breast Cancer, Discipline of Integrated Chinese and Western Medicine, The second affiliated hospital of Guangzhou University of Chinese Medicine, Guangzhou, Guangdong, China
[3]Post-Doctoral Research Center, Guangzhou University of Chinese Medicine, Guangzhou, Guangdong, China;
[4]School of Chinese Medicine, the University of Hong Kong, Hong Kong SAR

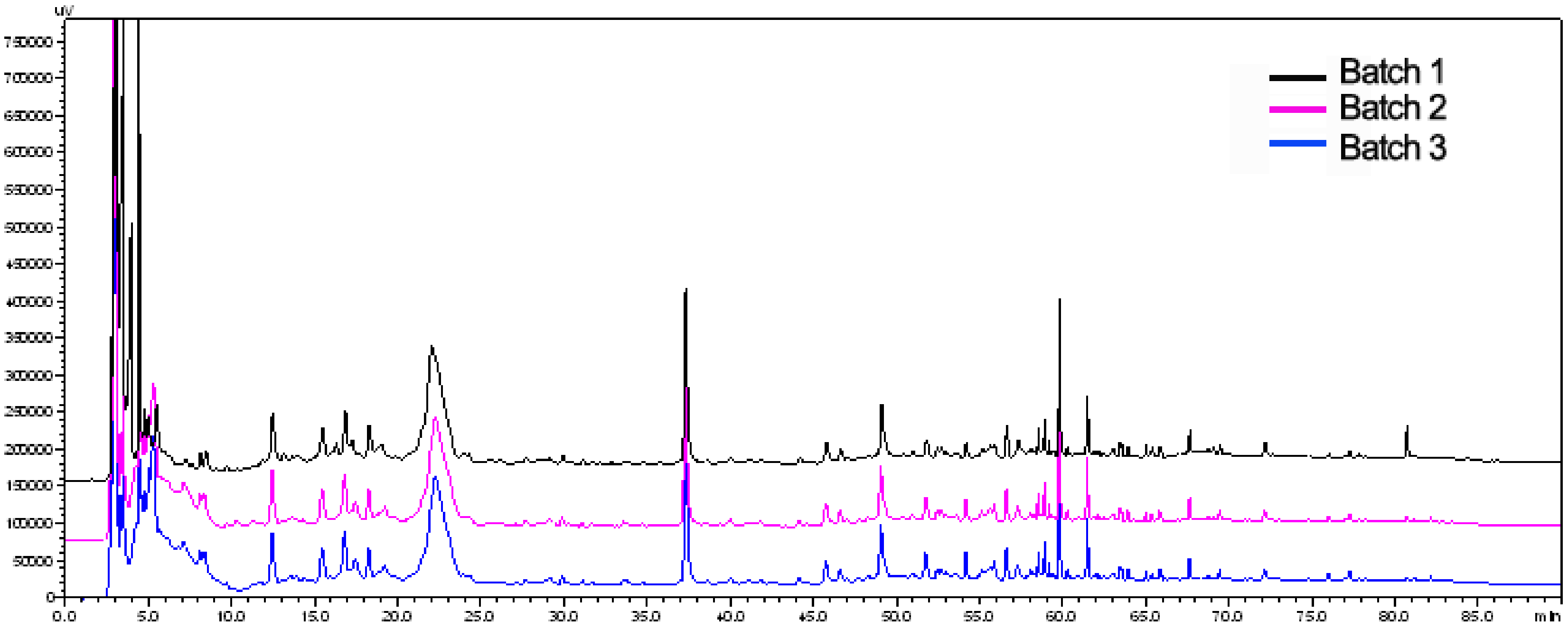

Supplementary Figure 1

Supplementary Figure 1 Chemical fingerprints remains consistent between 3 batches of XIOPI formula.

	A	B	C	D	E	F	G	H	I	J	K	L
1	POS	POS	NEG	NEG	6Ckine	CTACK	Eotaxin-1	G-CSF	GM-CSF	IL-2	IL-3	IL-4
2	POS	POS	NEG	NEG	6Ckine	CTACK	Eotaxin-1	G-CSF	GM-CSF	IL-2	IL-3	IL-4
3	IL-5	IL-6	IL-9	IL-10	IL12p40/70	IL-12p70	IL-13	IL-17	IFN gamma	KC	Leptin	MCP-1
4	IL-5	IL-6	IL-9	IL-10	IL12p40/70	IL-12p70	IL-13	IL-17	IFN gamma	KC	Leptin	MCP-1
5	MCP-5	MIP-1 alpha	MIP-2	MIP-2 beta	RANTES	SCF	sTNFRI	TARC	TIMP-1	TNF alpha	THPO	VEGF
6	MCP-5	MIP-1 alpha	MIP-2	MIP-2 beta	RANTES	SCF	sTNFRI	TARC	TIMP-1	TNF alpha	THPO	VEGF
7	BLANK	BLANK	BLANK	BLANK	BLANK	BLANK	BLANK	BLANK	BLANK	BLANK	BLANK	POS
8	BLANK	BLANK	BLANK	BLANK	BLANK	BLANK	BLANK	BLANK	BLANK	BLANK	BLANK	POS

Supplementary Figure 2 Cytokine layout of the mouse cytokine antibody array C2 kits.

Supplementary Table 1: The putative major ingredients and targets of 10 herbs in XIAOPI formula

Latin name	Chinese Name	Number of Screening ingredients	Major ingredients	Number of Targets
**Epimedium Brevicornum**	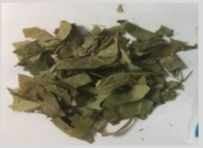 淫羊藿	**23**	24-epicampesterol<br>Linoleyl acetate<br>poriferast-5-en-3beta-ol<br>DFV<br>Chryseriol<br>8-Isopentenyl-kaempferol<br>sitosterol<br>kaempferol<br>olivil<br>Anhydroicaritin<br>C-Homoerythrinan, 1,6-didehydro-3,15,16-trimethoxy-, (3.beta.)-<br>Yinyanghuo A<br>Yinyanghuo C<br>Yinyanghuo E<br>6-hydroxy-11,12-dimethoxy-2,2-dimethyl-1,8-dioxo-2,3,4,8-tetrahydro-1H-isochromeno[3,4-h]isoquinolin-2-ium<br>8-(3-methylbut-2-enyl)-2-phenyl-chromone	**246**

			Anhydroicaritin-3-O-alpha-L-rhamnoside<br>1,2-bis(4-hydroxy-3-methoxyphenyl)propan-1,3-diol<br>Icariin<br>Icariside A7<br>luteolin<br>Magnograndiolide<br>quercetin	
**Cistanche Deserticola**	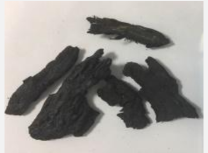<br>肉苁蓉	**6**	beta-sitosterol<br>Marckine<br>arachidonate<br>quercetin<br>suchilactone<br>Yangambin	**198**
**Ligustrum Lucidum**	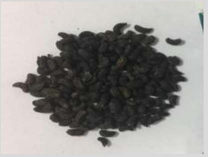<br>女贞子	**9**	beta-sitosterol<br>kaempferol<br>taxifolin<br>Lucidumoside D<br>Lucidumoside D_qt<br>eriodictyol<br>Olitoriside_qt<br>luteolin<br>quercetin	**215**
			1,2,5,6-tetrahydrotanshinone<br>Poriferasterol<br>poriferast-5-en-3beta-ol<br>isoimperatorin	

**Salvia Miltiorrhiza**	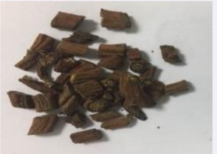<br>丹参	**38**	sugiol<br>Dehydrotanshinone II A<br>5,6-dihydroxy-7-isopropyl-1,1<br>-dimethyl-2,3-dihydrophenanthren-4-one<br>2-isopropyl-8-methylphenanthrene-3,4-dione<br>3α-hydroxytanshinoneⅡa<br>4-methylenemiltirone<br>formyltanshinone<br>Methylenetanshinquinone<br>przewaquinone c<br>sclareol<br>tanshinaldehyde<br>Danshenol B<br>Salvilenone<br>cryptotanshinone<br>dan-shexinkum d<br>danshenspiroketallactone<br>deoxyneocryptotanshinone<br>dihydrotanshinlactone<br>dihydrotanshinoneⅠ<br>epidanshenspiroketallactone<br>C09092<br>isocryptotanshi-none<br>Isotanshinone II<br>manool<br>miltionone Ⅱ	**115**

			miltipolone<br>Miltirone<br>neocryptotanshinone ii<br>1-methyl-8,9-dihydro-<br>7H-naphtho[5,6-g]benzofuran-6,10,11-trione<br>salvilenone Ⅰ<br>salviolone<br>tanshinone iia<br>(6S)-6-(hydroxymethyl)-1,6-dimethyl-8,9-dihydro-<br>7H-naphtho[8,7-g]benzofuran-10,11-dione<br>tanshinone Ⅵ	
**Curcuma Aromatica**	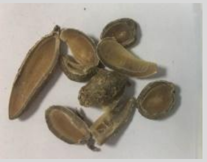<br>郁金	**3**	beta-sitosterol<br>Sitosterol<br>Naringenin	**69**
**Rhizoma Curcumae**	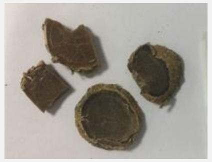<br>莪术	**3**	hederagenin<br>Germacron<br>curzerene	**32**
			galeopsin<br>ZINC04073977<br>preleoheterin	

**Leonurus heterophyllus**	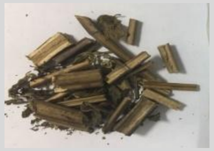<br>益母草	**8**	iso-preleoheterin<br>quercetin<br>arachidonic acid<br>isorhamnetin<br>kaempferol	**223**
**Radix Polygoni Multiflori Preparata**	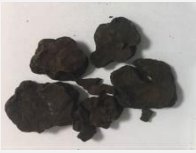<br>何首乌	**6**	questin<br>rhein<br>emodin<br>polygodial<br>chrysazin<br>resveratrol	**358**
**Crassostrea Gigas**	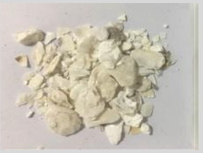<br>牡蛎	**5**	aluminum<br>calcium sulphate<br>calcium phosphate<br>silicon<br>calcium carbonate	**149**
**Carapax Trionycis**	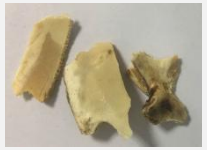<br>鳖甲	**4**	gondoic acid<br>Vaccenic acid<br>8-Octadecenoic acid<br>oleic acid	**182**

Supplementary Table 2 The targeting genes of XIAOPI formula related to breast cancer

COL1A1 POSTN EGFR SPARC FN1 TF CXCL10 MMP2 STAT1 CCNA2 NCOA3 IFI27 E2F1 PLAT JUN TGFA CAV1 HSPA5 BIRC5 BMP2 FAP PCNA FOS TGFB2 PDGFB INSR TNFRSF10B CDK2 NCOA1 GSTP1 CTSK PIK3CB CA2 CHEK1 NR1I2 TOP2A GSTM1 PPARA CAT CD80 EGR1 PARP1 IL2RA EDNRA CCNE1 VEGFA CDK1 PDGFRB BCL2 GNAS MMP1 CCNB1 RAD51HIF1A G6PD PTGS1 MAP2K1 CXCL1 ROCK1 FGF1 SPP1 PLAU VCAM1 BRCA2 ERCC1 PTGS2 CDKN2A EGF TNS1 PRKAA1 TNFRSF10A IRS1 MAPK14 CCNE2 GDF15 PTK2 CLDN4 CD28 SLC6A3 CFLAR BRCA1
